# Nutritional factors for anemia in pregnancy: A systematic review with meta-analysis

**DOI:** 10.3389/fpubh.2022.1041136

**Published:** 2022-10-14

**Authors:** Jing Zhang, Quanhong Li, Ying Song, Liping Fang, Lei Huang, Yu Sun

**Affiliations:** ^1^Department of Obstetrics, Kunming City Maternal and Child Health Hospital, Kunming, China; ^2^Kunming Municipal Service Center for Maternal and Child Health, Kunming, China; ^3^Kunming Children's Hospital, Kunming, China; ^4^Department of Pharmacy, Children's Hospital of Kunming Medical University, Kunming, China

**Keywords:** anemia, pregnancy, systematic review, nutritional factors, evidence

## Abstract

**Background:**

Anemia in pregnancy is a serious threat to maternal and child health and is a major public health problem. However, the risk factors associated with its incidence are unclear and controversial.

**Methods:**

PubMed, Ovid Embase, Web of Science, and Cochrane databases were systematically searched (inception to June 27, 2022). The screening of search results, extraction of relevant data, and evaluation of study quality were performed independently by two reviewers.

**Results:**

A total of 51 studies of high quality (NOS score ≥ 7) were included, including 42 cross-sectional studies, six case-control studies, and three cohort studies. Meta-analysis showed that infected parasite, history of malarial attack, tea/coffee after meals, meal frequency ≤ 2 times per day, frequency of eating meat ≤ 1 time per week, frequency of eating vegetables ≤ 3 times per week, multiple pregnancies, multiparous, low household income, no antenatal care, rural residence, diet diversity score ≤ 3, have more than 3 children, history of menorrhagia, underweight, family size ≥ 5, middle upper arm circumference < 23, second trimester, third trimester, birth interval ≤ 2 year were all risk factors for anemia in pregnancy.

**Conclusions:**

Prevention of anemia in pregnancy is essential to promote maternal and child health. Sufficient attention should be paid to the above risk factors from the social level and pregnant women's own aspects to reduce the occurrence of anemia in pregnancy.

**Systematic review registration:**

https://www.crd.york.ac.uk/prospero/, identifier: CRD42022344937.

## Background

As a global public health problem, anemia in pregnancy has been shown to be an independent risk factor for adverse maternal and infant outcomes such as blood transfusion, postpartum hemorrhage, cesarean section, hysterectomy, preterm birth, and infectious diseases (1). It directly threatens the health of about 32 million pregnant women around the world. Especially in developing countries, 56% of pregnant women are affected by it (2, 3). Anemia in pregnancy is a global concern as it impairs physical health, cognitive development, productivity, and reflects lagging economic status (2, 4). Improving anemia in pregnancy is essential to reduce maternal and infant mortality and serious complications. Unfortunately, even though extensive studies have been conducted over the past 20 years and various national nutrition programs have been implemented to reduce anemia in pregnancy, there has not been much success in eliminating anemia in pregnancy, and it remains a major public health problem (4, 5).

It is critical to explore the risk factors that may cause anemia in pregnancy and take preventive strategies as soon as possible. However, the risk factors for anemia in pregnancy are controversial. For example, the findings of Kedir et al. suggest that parasite infection is not a risk factor for anemia in pregnancy (6). However, other studies in the same area identified parasitic infection as a risk factor for anemia in pregnancy (7, 8). It has also been shown that tea/coffee after meals is not a risk factor for anemia in pregnancy (AOR = 1.03, 95% CI: 0.88–2.06) (9), but the results of Teshome et al. showed a very significant association between tea/coffee after meals and anemia in pregnancy (AOR = 18.49, 95% CI: 6.89–40) (10). In addition, iron deficiency is considered to be the most common cause of anemia in pregnancy, therefore, most studies recommend that pregnant women should take adequate iron supplements to prevent anemia in pregnancy (11, 12). On the contrary, some studies have shown that iron supplementation did not reduce the incidence of anemia in pregnancy (13, 14). Some studies have even concluded that even without iron supplementation during pregnancy, the incidence of anemia in pregnant women is not significantly higher (15). In conclusion, disparate findings on the same exposure factors pose an obstacle to the prevention of anemia in pregnancy and further public health decisions.

The current field lacks definitive evidence on the risk factors for anemia in pregnancy. Therefore, as the first study to systematically summarize the risk factors of anemia in pregnancy, the results of this study can provide a reference for the prevention and treatment of anemia in pregnancy in the future.

## Methods

This systematic review and meta-analysis followed the Preferred Reporting Items for Systematic Reviews and Meta-Analyses (PRISMA) guidelines (16). The review protocol has been registered with PROSPERO, number CRD42022344937 (https://www.crd.york.ac.uk/prospero/).

### Inclusion and exclusion criteria

#### Patients and diseases (P)

Pregnant women.

#### Interventions (I)

Report at least one exposure factor associated with anemia in pregnancy.

#### Control (C)

Studies where adjusted odds ratio (AOR) for exposure factors were available or calculated.

#### Outcome (O)

Anemia in pregnancy occurs. The diagnostic criteria are hemoglobin ≤ 11 g/dL.

#### Type of study (S)

Cross-sectional studies, case-control studies, and cohort studies.

#### Exclusion criteria

Animal studies and cell experiments were excluded. Reviews, case reports, opinion articles, conference abstracts, and non-published data were also excluded.

### Data sources and searches

Candidate studies were identified through searches of the PubMed, Web of Science, Cochrane, and Ovid Embase databases from inception until June 27, 2022. Also, the reference lists of the included studies were searched. The retrieval approach of the combination of free words and subject words was adopted. The following terms were combined to generate search keywords: [gestational anemia OR anemia in pregnancy OR (pregnancy OR pregnant OR gestation) AND anemia] AND (hazard OR risk factors OR risk factor OR related factors OR factors OR influence factors OR influencing factors). Further details of the search strategy are shown in Supplementary Table 1.

### Data extraction and risk of bias assessment

Literature screening and data extraction were performed by 2 trained researchers according to the inclusion and exclusion criteria as indicated previously. Extracted content includes: (1) Basic information of included studies: authors, year, country, type of study, sample size, age, method of obtaining information, diagnostic criteria for anemia, and data analysis methods. (2) Exposure factors: Risk factors related to dietary habits, self-condition, and disease history of pregnant women. (3) Key elements of risk of bias assessment.

Based on the Newcastle-Ottawa Scale (NOS), two qualified researchers independently evaluated the inherent risk of bias of included studies from three aspects, including the selection of participants, confounding variables, and measurement of exposure (17). The evaluation results were scored as low, medium, and high quality, respectively, with scores of 0–3, 4–6, and 7–9.

### Statistical analysis

Statistical analysis was performed using STATA 16 software. Results were calculated using adjusted odds ratio (AOR) and 95% confidence interval (95% CI). The χ^2^ test was used to evaluate the heterogeneity of the included studies (the test level was α = 0.05), and the size of the heterogeneity was judged according to the *I*^2^ value. When *P* > 0.05 and *I*^2^ ≤ 50%, it indicated that the heterogeneity of the results of each study was not statistically significant, and a fixed-effects model was used for meta-analysis; otherwise, after further analysis of the source of heterogeneity, a random-effects model was used.

## Results

### Literature screening results

**Figure 1:**
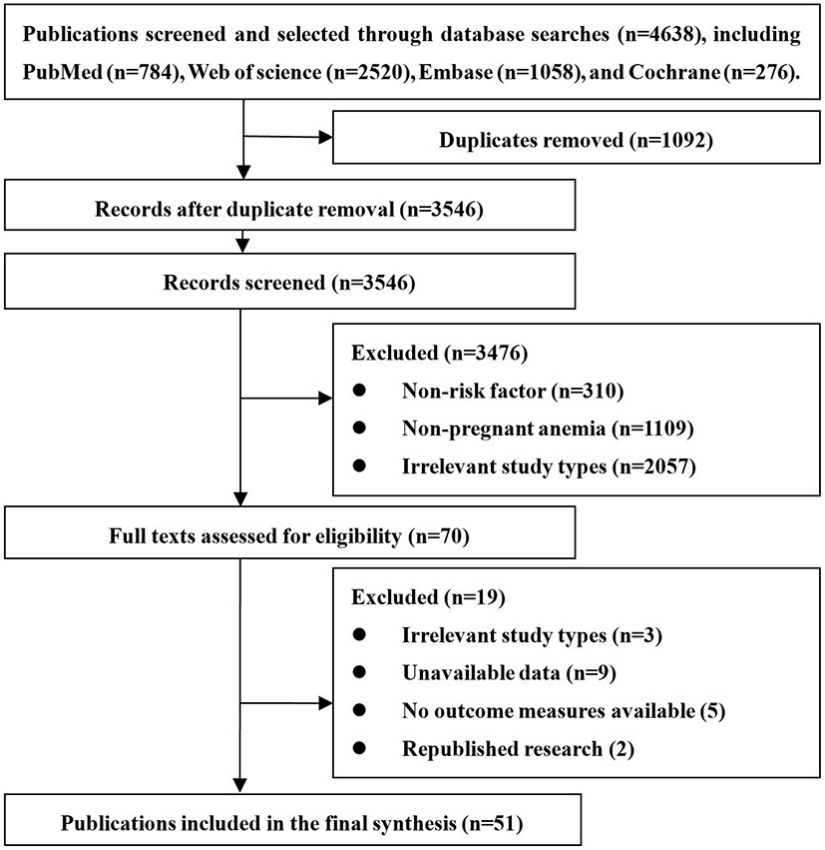
Flow diagram for study inclusion.

A total of 4,638 relevant records were obtained from the initial inspection, which were excluded from repeated studies, non-risk factor studies (prevalence, diagnosis, and treatment of anemia in pregnancy), pregnant women without anemia (postpartum anemia, women of childbearing age) and study types that are not consistent (review, conference summary, case report, etc.), 51 studies were finally included. The article screening process is shown in Figure 1.

### Basic information and risk of bias assessment of included studies

A total of 51 studies were included (6–10, 13–15, 18–60), including 42 cross-sectional studies (6–9, 13–15, 18–29, 31–37, 39–43, 45, 47–49, 51, 53, 54, 56–58, 60), six case-control studies (10, 30, 38, 50, 52, 55), and three cohort studies (44, 46, 59). The entire population was from developing countries, and 36 study sites were in Ethiopia (6–10, 13–15, 18–20, 23, 26–31, 34–43, 50, 52, 55–60). The total number of patients was 73,919 and in individual study ranged from 163 (49)−12,403 (53). The patients were aged between 15 and 49 years (40). Information was obtained through structured questionnaires (6–10, 13–15, 18–39, 41–45, 47–50, 52–56, 59, 60), outpatient medical record data (40, 46, 51, 58), and databases (57). The diagnostic criteria for anemia were hemoglobin < 11 g/dL, and the statistical analysis methods were multivariable logistic regression. See Table 1 for details.

**Table 1:** Basic information of included studies.

**References**	**Country**	**Study design**	**Sample size**	**Age**	**Factors**
Kedir et al. (6)	Ethiopia	Cross-sectional study	284	27.2 ± 4.9	Meal frequency ≤ 2 times per day; Frequency of eating meat ≤ 1 time per week; History of menorrhagia; Low household income; Second trimester; Third trimester; Birth interval ≤ 2 year; Lack of understanding of anemia; Illiteracy; Infected parasite; History of malarial attack.
Balis et al. (9)	Ethiopia	Cross-sectional study	456	/	Tea/coffee after meals; History of menorrhagia; Low household income; Rural residence; Rural residence; Birth interval ≤ 2 year; Have more than 3 children; Abortion history.
Abdella et al. (14)	Ethiopia	Cross-sectional study	241	26.9 ± 6.3	Frequency of eating meat ≤ 1 time per week; Iron supplementation; Family size ≥ 5; Low household income; Rural residence; Illiteracy; Have more than 3 children.
Abdu et al. (18)	Ethiopia	Cross-sectional study	314	26 ± 2.15	Frequency of eating vegetables ≤ 3 times per week; History of menorrhagia; Underweight; Rural residence; Birth interval ≤ 2 year; No antenatal care; Middle upper arm circumference < 23.
Abriha et al. (19)	Ethiopia	Cross-sectional study	619	27.4 ± 5.5	Meal Frequency ≤ 2 times per day; Frequency of eating meat ≤ 1 time per week; Diet Diversity Score ≤ 3; Have more than 3 children.
Addis Alene and Mohamed Dohe (20)	Ethiopia	Cross-sectional study	577	27.01 ± 5.97	No iron supplementation; Second trimester; Third trimester; Have more than 3 children.
Alreshidi and Haridi (21)	Saudi Arabia	Cross-sectional study	390	29.9 ± 7.56	Tea/coffee after meals; Frequency of eating meat ≤ 1 time per week; History of menorrhagia; Family size≥5; Low household income.
Anwary et al. (22)	Afghanistan	Cross-sectional study	787	30.48 ± 7.02	Age ≥ 35 years old; Multiple pregnancies; Rural residence.
Asrie (23)	Ethiopia	Cross-sectional study	206	28.34 ± 6.1	Rural residence; HIV status; Infected parasite.
Nonterah et al. (24)	Ghana	Cross-sectional study	506	20–34	Age ≥ 35 years old; Underweight; Overweight; Obesity; No antenatal care.
Azhar et al. (25)	Bangladesh	Cross-sectional study	424	16–40	Multiple pregnancies; Multiparous; Low household income.
Bekele et al. (26)	Ethiopia	Cross-sectional study	332	25 ± 4.28	Family size ≥ 5; Birth interval ≤ 2 year.
Berhe et al. (27)	Ethiopia	Cross-sectional study	304	25.3 ± 5.1	No iron supplementation; History of menorrhagia; Rural residence; Third trimester; Abortion history.
Berhe et al. (28)	Ethiopia	Case-control study	600	17–40	Tea/coffee after meals; Diet Diversity Score ≤ 3; Rural residence; Diet Diversity Score ≤ 3; Infected parasite.
Beyene (29)	Ethiopia	Cross-sectional study	374	25.2 ± 5.28	Tea/coffee after meals; Frequency of eating vegetables ≤ 3 times per week; Have more than 3 children.
Debella et al. (13)	Ethiopia	Cross-sectional study	405	15–44	Tea/coffee after meals; Meal Frequency ≤ 2 times per day; Iron supplementation; Drinking; Rural residence; No antenatal care.
Deriba et al. (30)	Ethiopia	Case-control study	426	/	Tea/coffee after meals; Meal Frequency ≤ 2 times per day; Frequency of eating vegetables ≤ 3 times per week; Diet Diversity Score ≤ 3; Middle upper arm circumference < 23.
Derso et al. (31)	Ethiopia	Cross-sectional study	348	15–40	Meal Frequency ≤ 2 times per day; No iron supplementation; Low household income; Rural residence; Second trimester; Third trimester; Middle upper arm circumference < 23; Have more than 3 children.
Engmann et al. (32)	Ghana	Cross-sectional study	452	/	Third trimester; History of malarial attack.
Fondjo et al. (33)	Ghana	Cross-sectional study	628	28.44 ± 6.19	Second trimester; Third trimester; Have more than 3 children; History of malarial attack.
Gari et al. (34)	Ethiopia	Cross-sectional study	384	23.57 ± 3.8	Age ≥ 35 years old.
Gebre and Mulugeta (35)	Ethiopia	Cross-sectional study	714	25.8 ± 5.84	Meal frequency ≤ 2 times per day; No iron supplementation; Overweight; Low household income; Rural residence; Illiteracy.
Getachew et al. (8)	Ethiopia	Cross-sectional study	388	16–40	Rural residence; Infected parasite; History of malarial attack.
Grum et al. (36)	Ethiopia	Cross-sectional study	638	15–40	Meal Frequency ≤ 2 times per day; Unplanned pregnancy; Birth interval ≤ 2 year; History of malarial attack.
Gudeta et al. (37)	Ethiopia	Cross-sectional study	330	15–34	Tea/coffee after meals; No iron supplementation; Drinking; Age ≥ 35 years old; Multiparous; Family size ≥ 5; Rural residence; Third trimester; No antenatal care; Illiteracy; Middle upper arm circumference < 23; HIV status.
Hailu et al. (38)	Ethiopia	Cross-sectional study	743	15–35	Frequency of eating meat ≤ 1 time per week; Frequency of eating vegetables ≤ 3 times per week; Age ≥ 35 years old; Rural residence; Illiteracy; Infected parasite.
Helion Belay et al. (39)	Ethiopia	Cross-sectional study	713	18–40	Frequency of eating vegetables ≤ 3 times per week; No iron supplementation; Rural residence; Illiteracy.
Kare and Gujo (40)	Ethiopia	Cross-sectional study	340	15–49	History of menorrhagia; Low household income; First trimester; Illiteracy; Diet Diversity Score ≤ 3; HIV status; History of malarial attack.
Kebede et al. (41)	Ethiopia	Cross-sectional study	480	18–38	Birth interval ≤ 2 year; HIV status; Infected parasite.
Kefiyalew et al. (7)	Ethiopia	Cross-sectional study	258	26.9 ± 4.8	Age ≥ 35 years old; Rural residence; Second trimester; Third trimester; Illiteracy; Infected parasite.
Kejela et al. (15)	Ethiopia	Cross-sectional study	286	20–40	No iron supplementation; Age ≥ 35 years old; Birth interval ≤ 2 year; Infected parasite; History of malarial attack.
Kenea et al. (42)	Ethiopia	Cross-sectional study	416	25.15 ± 4.29	Underweight; Overweight; Multiple pregnancies; Family size ≥ 5; Rural residence; Birth interval ≤ 2 year; HIV status; Infected parasite; History of malarial attack.
Lebso et al. (43)	Ethiopia	Cross-sectional study	507	15–49	Frequency of eating meat ≤ 1 time per week; Diet Diversity Score ≤ 3; No iron supplementation; Low household income; Unplanned pregnancy; Second trimester; Third trimester; Illiteracy; Have more than 3 children; Infected parasite.
Lin et al. (44)	China	Cohort study	10,199	29.50 ± 4.46	Age ≥ 35 years old; Underweight; Overweight; Obesity; Low household income; Rural residence.
Ngimbudzi et al. (45)	Tanzania	Cross-sectional study	418	25 ± 6.83	Frequency of eating meat ≤ 1 time per week; Frequency of eating vegetables ≤ 3 times per week; Second trimester; Third trimester.
Noronha et al. (46)	India	Cohort study	1,077	17–30	History of menorrhagia; Underweight; Low household income; First trimester; Second trimester; Lack of understanding of anemia; Illiteracy.
Obai et al. (47)	Uganda	Cross-sectional study	743	19–39	Second trimester; Third trimester.
Oboro et al. (48)	Nigeria	Cross-sectional study	779	/	Second trimester; Third trimester; Have more than 3 children.
Okia et al. (49)	Uganda	Cross-sectional study	163	17–40	Drinking; History of menorrhagia; Low household income; HIV status.
Osman et al. (50)	Ethiopia	Case-control study	228	24.96 ± 5. 22	Frequency of eating meat ≤ 1 time per week; Frequency of eating vegetables ≤ 3 times per week; Illiteracy; Middle upper arm circumference < 23.
Ribot et al. (51)	Spain	Cross-sectional study	11,259	29.7 ± 5.7	Age ≥ 35 years old; Obesity; Multiple pregnancies; Low household income; Have more than 3 children.
Tadesse et al. (52)	Ethiopia	Case-control study	448	17–38	Frequency of eating meat ≤ 1 time per week; Frequency of eating vegetables ≤ 3 times per week; First trimester; Third trimester; HIV status.
Tan et al. (53)	China	Cross-sectional study	12,403	/	Age ≥ 35 years old; Underweight; Overweight; Multiple pregnancies; Second trimester; Third trimester.
Tan et al. (54)	China	Cross-sectional study	11,782	/	Underweight; Overweight; Obesity.
Teshome et al. (10)	Ethiopia	Case-control study	244	15–38	Tea/coffee after meals; History of menorrhagia; Rural residence; Infected parasite.
Tulu et al. (55)	Ethiopia	Case-control study	573	15–44	Diet Diversity Score ≤ 3; No iron supplementation; History of menorrhagia; Multiparous; Low household income; Birth interval ≤ 2 year; No antenatal care; Middle upper arm circumference < 23; Infected parasite; Abortion history.
Weldekidan et al. (56)	Ethiopia	Cross-sectional study	333	15–49	Tea/coffee after meals; Frequency of eating meat ≤ 1 time per week; History of menorrhagia; Birth interval ≤ 2 year; Infected parasite.
Woldegebriel et al. (57)	Ethiopia	Cross-sectional study	3,082	15 ± 1.5	Low household income; Illiteracy.
Yesuf and Agegniche (58)	Ethiopia	Cross-sectional study	286	25.7 ± 1.05	Frequency of eating vegetables ≤ 3 times per week; Rural residence; Infected parasite; History of malarial attack.
Zerfu et al. (59)	Ethiopia	Cohort study	432	20–40	Frequency of eating meat ≤ 1 time per week; Diet Diversity Score ≤ 3; Frequency of eating vegetables ≤ 3 times per week.
Zillmer et al. (60)	Ethiopia	Cross-sectional study	4,600	/	Iron supplementation; Middle upper arm circumference < 23.

The NOS scores of the 51 included studies were all ≥ 7 points, of which 38 studies had a NOS score of 8 points, and 13 studies had 7 points, indicating that the included studies had high research quality. See Supplementary Table 2 for details.

### Meta-analysis results

#### Exposure factors associated with maternal medical history

**Figure 2:**
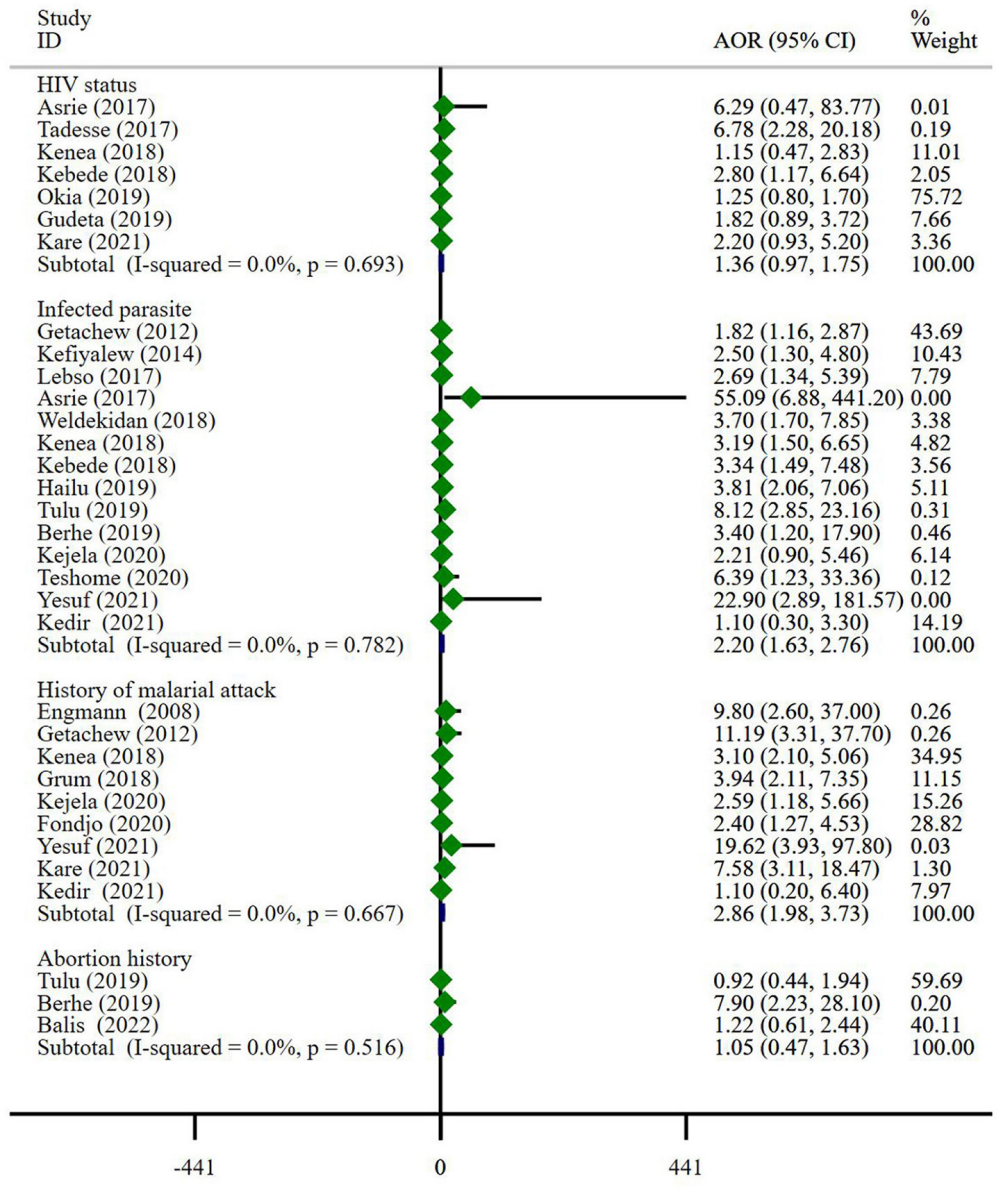
Exposure factors associated with maternal medical history.

A total of four exposure factors associated with medical history may contribute to anemia in pregnancy. Since *I*^2^ = 0, *P* > 0.05, indicating that there is little possibility of heterogeneity among the studies, a fixed-effect model was used for combined analysis. Meta-analysis showed that parasitic infection (AOR = 2.20, 95% CI: 1.63–2.76) and history of malarial attack (AOR = 2.86, 95% CI: 1.98–3.73) were risk factors for anemia in pregnancy, while HIV status (AOR = 1.36, 95% CI: 0.97–1.75) and abortion history (AOR = 1.05, 95% CI: 0.47–1.63) were not associated with anemia in pregnancy (Figure 2).

#### Exposure factors associated with dietary habits of pregnant women

A total of eight exposure factors related to dietary habits may contribute to anemia in pregnancy. The fixed effect model was used to analyze the exposure factors of *I*^2^ < 50%. The results showed that tea/coffee after meals, meal frequency ≤ 2 times per day, frequency of eating meat ≤ 1 time per week, diet diversity score ≤ 3 were risk factors for anemia in pregnancy. Iron supplementation was a protective factor for anemia in pregnancy (Table 2). Meta-analysis of the potential risk factors for *I*^2^ > 50 % using a random effect model showed that frequency of eating vegetables ≤ 3 times per week was a risk factor for anemia in pregnancy, while no iron supplementation and drinking were not associated with anemia in pregnancy (Table 2).

**Table 2:** Exposure factors associated with dietary habits of pregnant women.

**Exposure factors**	**Analytical model**	**Number of studies**	**Effect size**	**Heterogeneity**
Tea/coffee after meals	Fixed-effects model	9	1.63 (1.21, 2.04)	49.8%
Meal frequency ≤ 2 times per day		7	2.29 (1.61, 2.96)	0
Frequency of eating meat ≤ 1 time per week		11	2.02 (1.55, 2.50)	44.7%
Iron supplementation		3	0.79 (0.62, 0.96)	0
Diet diversity score ≤ 3		7	2.38 (1.55, 3.21)	42.3%
Frequency of eating vegetables ≤ 3 times per week	Random-effects model	10	2.97 (1.59, 4.34)	77.5%
No iron supplementation		9	1.38 (0.77, 1.99)	74.9%
Drinking		3	1.54 (0.37, 2.71)	55.4%

#### Exposure factors associated with maternal conditions

A total of 20 exposure factors associated with maternal conditions may contribute to anemia in pregnancy. Multiple pregnancies, multiparous, low household income, no antenatal care, rural residence, have more than 3 children, history of menorrhagia, underweight, family size ≥ 5, middle upper arm circumference < 23, second trimester, third trimester, and birth interval ≤ 2 year were all risk factors for anemia in pregnancy. Overweight was a protective factor, and the remaining exposure factors were not associated with anemia in pregnancy (Table 3).

**Table 3:** Exposure factors associated with maternal conditions.

**Exposure factors**	**Analytical model**	**Number of studies**	**Effect size**	**Heterogeneity**
Overweight	Fixed-effects model	6	0.72 (0.65, 0.79)	0.0%
Multiple pregnancies		5	1.65 (1.29, 2.01)	0.0%
Multiparous		3	1.50 (1.06, 1.95)	0.0%
Low household income		15	1.39 (1.23, 1.54)	45.8%
Unplanned pregnancy		2	1.64 (0.99, 2.29)	35.2%
First trimester		3	1.26 (0.93, 1.60)	0.0%
Lack of understanding of anemia		2	1.31 (0.95, 1.66)	0.0%
No antenatal care		5	2.02 (1.81, 2.22)	0.0%
Rural residence		19	1.40 (1.23, 1.57)	33.7%
Have more than 3 children		12	1.58 (1.28, 1.89)	28.1%
Age ≥ 35 years old	Random-effects model	10	1.15 (0.86, 1.43)	67.5%
History of menorrhagia		11	2.11 (1.35, 2.86)	51.7%
Underweight		7	1.23 (1.04, 1.42)	57.9%
Obesity		4	0.53 (0.35, 0.72)	56.2%
Family size ≥ 5		5	1.99 (1.01, 2.96)	55.7%
Middle upper arm circumference < 23		7	2.75 (1.60, 3.90)	82.2%
Second trimester		12	1.84 (1.36, 2.31)	50.6%
Third trimester		14	2.20 (1.43, 2.98)	77.8%
Birth interval ≤ 2 year		10	2.84 (1.59, 4.09)	87.9%
Illiteracy		12	1.34 (0.92, 1.76)	66.6%

### Publication bias

**Figure 3:**
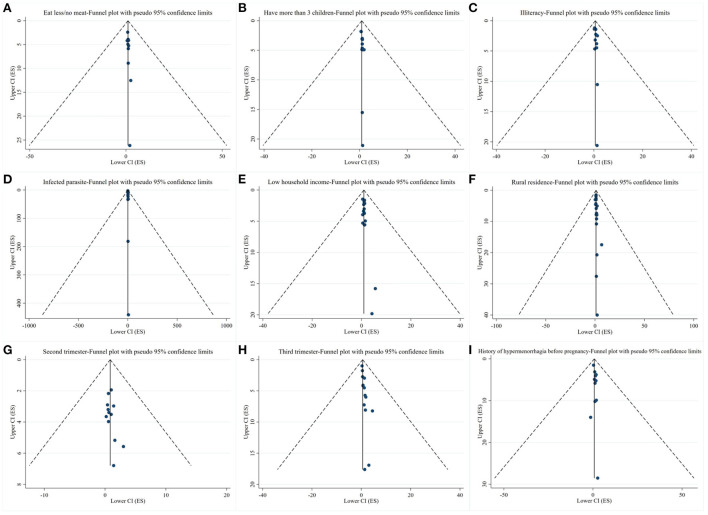
Results of publication bias. **(A–I)** Funnel plots of exposure factors with more than 10 studies.

Funnel plots were drawn for exposure factors with more than 10 studies to detect publication bias. The results showed that the funnel plots were basically symmetrical, suggesting a small possibility of publication bias (Figure 3).

## Discussion

### Summary of evidence related to maternal medical history

Due to poverty, lack of safe drinking water, poor hygiene, and malnutrition, combined with the immunomodulatory and physiological changes that occur during pregnancy, pregnant women are often more vulnerable than non-pregnant women to intestinal parasite invasion, especially in developing countries (61). More than 7 million pregnant women worldwide are infected with hookworm, and 10 million pregnant women in Africa are infected with schistosomiasis (62, 63). Parasites entering the gut can attach to the mucosa and submucosa of the small intestine, destroy capillaries and arterioles and feed on the exuding blood (64). Our findings suggest that parasitic infection is one of the risk factors for anemia in pregnancy. This finding is consistent with the study conducted by Alem et al. (65). In developing countries, infection of young women, pregnant women, and their infants with intestinal parasites, especially hookworms, can lead to deficiencies in iron, total energy, protein, and folic acid and zinc, leading to low birth weight, intrauterine growth retardation, and higher morbidity and mortality of anemia in pregnancy (58). Moreover, as another common risk factor, infection with malaria is also susceptible to anemia in pregnancy. Notably, studies on malaria have come from both Ethiopia and Ghana. Their geographical location in the tropics has an important impact on the distribution of malaria. Sequestration of plasmodium in the placenta avoids spleen clearance, thereby predisposing pregnant women to malaria. Malaria causes anemia in a variety of ways, including excessive depletion of non-parasitic red blood cells, immune destruction of parasitic red blood cells, and impaired erythropoiesis due to bone marrow dysfunction (66, 67). Other studies have shown that pregnant women infected with HIV are more likely to develop anemia than those who are not infected with HIV (41). This may be due to the properties of the virus that lead to increased metabolic and nutritional requirements and directly inhibit the production of red blood cells in the body (68). Although our meta-analysis showed no significant association between HIV infection and anemia in pregnancy. However, the lower limit of confidence interval of our results is close to 1, suggesting to some extent the correlation between HIV infection and anemia in pregnancy.

### Summary of evidence related to maternal status

Previous study has shown that the risk of anemia in pregnancy increases with the number of births. The risk of developing anemia in pregnancy was nearly 3 times higher for women with 2–3 children and 4 times higher for women with 4 or more children compared to only one child (69, 70). This is because pregnant women do not have enough time to recover from the nutritional burden of their previous pregnancy, especially folic acid, and iron deficiency. Maternal serum and erythrocyte folate concentrations also decline from the fifth month of pregnancy and remain low for a considerable time after delivery (6). The same is true for our combined analysis of 12 studies, finding that women with more than 3 children were more likely to develop anemia in pregnancy. In addition, multiple pregnancies, multiparous, and birth interval ≤ 2 years are also risk factors for anemia in pregnancy. Like the reasons for having more children, these factors lead to impaired iron stores in pregnant women, and to a certain extent, they impair the normal physiological functions and anatomical structures of pregnant women. Studies have shown that during pregnancy, the incidence of anemia increases more than 4 times from the first trimester to the third trimester, and the prevalence in the third trimester is as high as 30–45% (71). This is consistent with our findings that early pregnancy is less prone to anemia, whereas second and third trimesters are significantly associated with anemia. It may be related to the rapid growth of the fetus in the second and third trimesters and the significant increase in the demand for nutrients such as iron (72).

According to the World Health Organization, anemia in pregnancy is more prevalent in developing countries, such as Africa and Southeast Asia, where dietary diversity, living standards, and education levels are all poorer (73). In addition, lack of knowledge about anemia, infrequent antenatal check-ups, and unplanned pregnancies naturally lead to more threats of anemia in local pregnant women (74). As our study shows, 51 studies are from developing countries, especially Ethiopia, Ghana, and other countries. Also, low household income, no antenatal care, rural residence, underweight, middle upper arm circumference < 23, and illiteracy are all risk factors for anemia in pregnancy. Although unplanned pregnancy and lack of understanding of anemia were not statistically associated with anemia in pregnancy. However, according to the OR value of more than 1, and the lower limit of the 95% confidence interval close to 1, it is suggested that these two exposure factors are related to anemia in pregnancy to a certain extent.

### Summary of evidence related to dietary habits in pregnant women

In fact, the most common cause of anemia in pregnancy is iron deficiency, while other causes are rare (2). Although our study shows that lack of iron supplementation during pregnancy is not associated with anemia, the likely reason is that pregnant women obtain adequate iron intake through other means such as diet. However, our study also shows that iron supplementation is a protective factor in reducing the occurrence of anemia. This fully illustrates the importance of ingesting or supplementing adequate iron during pregnancy. Adequate intake of macro- and micronutrients, quantity and variety of diets is a challenge in many countries, especially developing ones (75, 76). After combined analysis of exposure factors related to dietary habits of pregnant women, we found that meal frequency ≤ 2 times per day, frequency of eating meat ≤ 1 time per week, tea/coffee after meals, diet diversity score ≤ 3, frequency of eating vegetables ≤ 3 times per week were all risk factor for anemia in pregnancy. This is consistent with the findings of Roess et al. Tea and coffee contain compounds that affect iron absorption such as tannins and polyphenol, meat is a good source of heme iron and protein of high biological value, and fruits rich in ascorbic acid can enhance iron absorption (77). Therefore, eating less or not eating meat and fruits will also lead to insufficient iron intake, which will eventually lead to the occurrence of anemia (78, 79). In addition, vegetables are a food source of folic acid, and folic acid deficiency is associated with anemia in pregnancy (80).

Although anemia in pregnancy is a global public health problem, we must acknowledge that anemia in pregnancy often differs between developed and developing countries and is one of the distinct health disparities between developed and developing countries (81). The prevalence of anemia in pregnancy in developing countries ranged between 53.8 and 90.2%, compared with 8.3% in developed countries (82). There are many factors that contribute to this difference. Compared with developed countries, medical resources are scarce in developing countries, pregnant women are less likely to receive adequate or quality health care, and they are at higher risk of exposure to diseases such as malaria and parasitic infections that cause anemia in pregnancy (83). Although we conducted a comprehensive search of current mainstream databases, the studies we included were all from developing countries, and evidence from developed countries was lacking. Therefore, our findings are only applicable to developing countries. More findings from developed countries are needed in the future to provide a global picture of risk factors for anemia in pregnancy.

### Strengths and limitations

Strengths: (1) As the first study in the current field to systematically summarize the risk factors of anemia in pregnancy, this study may serve as the best evidence for the prevention of anemia in pregnancy in the future. (2) This study was based on AOR rather than OR analysis, avoiding the interaction between multiple exposure factors, and the results were more in line with the actual situation. (3) According to the NOS scoring results, we found that the quality of evidence of the 51 included studies was high, which ensured the reliability of the meta-analysis results.

Limitations: (1) The included studies were all from developing countries, especially Ethiopia, therefore, our findings are only applicable to some countries, not all countries. (2) Include only English literature, which may lead to language bias. (3) The gray literature and conference abstracts were not searched, which may lead to publication bias.

## Conclusions

The high incidence and serious harm of anemia in pregnancy make it urgent to systematically summarize its risk factors. Evidence from 51 high-quality studies showing infected parasite, history of malarial attack, tea/coffee after meals, meal frequency ≤ 2 times per day, frequency of eating meat ≤ 1 time per week, frequency of eating vegetables ≤ 3 times per week, multiple pregnancies, multiparous, low household income, no antenatal care, rural residence, diet diversity score ≤ 3, have more than 3 children, history of menorrhagia, underweight, family size ≥ 5, middle upper arm circumference < 23, second trimester, third trimester, birth interval ≤ 2 year, these 20 exposure factors were all risk factors for Anemia in Pregnancy. Therefore, health institutions and pregnant women themselves should focus on the above risk factors for better prevention and early detection of anemia in pregnancy.

## Data availability statement

The original contributions presented in the study are included in the article/Supplementary material, further inquiries can be directed to the corresponding author/s.

## Author contributions

YSu proposed ideas and designed protocol. JZ and QL were responsible for data analysis and writing of the paper. YSo, LF, and LH were responsible for literature screening, data extraction, and quality evaluation. All authors contributed to the article and approved the submitted version.

## Funding

We acknowledge the financial support from Kunming Health Science and Technology Personnel Training Project (No. 2022-SW-93).

## Conflict of interest

The authors declare that the research was conducted in the absence of any commercial or financial relationships that could be construed as a potential conflict of interest.

## Publisher's note

All claims expressed in this article are solely those of the authors and do not necessarily represent those of their affiliated organizations, or those of the publisher, the editors and the reviewers. Any product that may be evaluated in this article, or claim that may be made by its manufacturer, is not guaranteed or endorsed by the publisher.

## Abbreviations

PRISMA: Preferred Reporting Items for Systematic Reviews and Meta-Analyses
AOR: adjusted odds ratio
NOS: Newcastle-Ottawa Scale.
