# Supplementary material for: Nutritional factors for anemia in pregnancy: A systematic review with meta-analysis
Source: Front Public Health. 2022 Oct 14;10:1041136. doi: 10.3389/fpubh.2022.1041136 (PMC9615144; doi:10.3389/fpubh.2022.1041136)
Supplement: Supplementary file 1 [file Data_Sheet_1.docx]

Supplementary Material

**Table 1: Literature search strategy**

| 1.Pubmed  #1:"Pregnancy"[MeSH Terms] AND "Anemia"[MeSH Terms] 15605  #2: "gestational anemia"[Title/Abstract] OR "anemia during pregnancy"[Title/Abstract] OR (("pregnancy"[Title/Abstract] OR "pregnant"[Title/Abstract] OR "gestation"[Title/Abstract]) AND "anemia"[Title/Abstract]) 7958  #3: #1 OR #2 9,864  #4: "Risk Factors"[MeSH Terms] 933568  #5: "hazard"[Title/Abstract] OR "risk factors"[Title/Abstract] OR "risk factor"[Title/Abstract] OR "related factors"[Title/Abstract] OR "factors"[Title/Abstract] OR ((("influence"[All Fields] OR "influenced"[All Fields] OR "influences"[All Fields] OR "influencing"[All Fields]) AND ("factor"[All Fields] OR "factor s"[All Fields] OR "factors"[All Fields])) AND "influencing factors"[Title/Abstract]) 2,737,304  #6: #4 OR #5 3,204,784  #7: #3 AND #6 784  2.WOS  (TS=(hazard OR ‘risk factors’ OR ‘risk factor’ OR ‘related factors’ OR factors OR ‘influence factors’ OR ‘influencing factors’)) AND TS=(‘gestational anemia’ OR ‘anemia during pregnancy’ OR ((pregnancy OR pregnant OR gestation) AND anemia)) 2520  3.embase  #1: 'gestational anemia':ab,ti OR 'anemia during pregnancy':ab,ti OR ((pregnancy:ab,ti OR pregnant:ab,ti OR gestation:ab,ti) AND anemia:ab,ti) 11120  #2: 'pregnancy'/exp 844921  #3: 'gestation'/exp 844921  #4: 'anemia'/exp 452725  #5: #2 OR #3 844921  #6: #4 AND #5 25608  #7: #1 OR #6 30654  #8: hazard:ab,ti OR 'risk factors':ab,ti OR 'risk factor':ab,ti OR 'related factors':ab,ti OR factors:ab,ti OR 'influence factors':ab,ti OR 'influencing factors':ab,ti 3,588,524  #9: 'hazard'/exp 785,607  #10: 'risk factors'/exp 1,239,486  #11: 'risk factor'/exp 1,239,486  #12: #8 OR #9 OR #10 OR #11 4,537,600  #13: #7 AND #12 1058  4.Cochrane  #1: (‘gestational anemia’ OR ‘anemia during pregnancy’ OR ((pregnancy OR pregnant OR gestation) AND anemia)):ti,ab,kw 2372  #2: MeSH descriptor: [Pregnancy] explode all trees 24735  #3: MeSH descriptor: [Anemia] explode all trees 5771  #5: #2 AND #3 468  #6: #1 OR #4 2411  #7: (hazard OR ‘risk factors’ OR ‘risk factor’ OR ‘related factors’ OR factors OR ‘influence factors’ OR ‘influencing factors’):ti,ab,kw 300981  #8: MeSH descriptor: [Risk Factors] explode all trees 26311  #9: #6 OR #7 300981  #10: #5 AND #8 276 |
| --- |

**Table 2:** **Risk of bias assessment results**

| **Author** | **Year** | **The selection of participants** | **Confounding variables** | **Measurement of exposure** | **Total score** |
| --- | --- | --- | --- | --- | --- |
| Yesuf | 2021 | 4 | 2 | 2 | 8 |
| Ribot | 2018 | 4 | 2 | 2 | 8 |
| Kare | 2021 | 4 | 2 | 1 | 7 |
| Tan | 2018 | 4 | 2 | 2 | 8 |
| Tsegay | 2018 | 4 | 2 | 2 | 8 |
| Kefiyalew | 2014 | 4 | 2 | 2 | 8 |
| Abriha | 2014 | 4 | 2 | 2 | 8 |
| Getachew | 2012 | 4 | 2 | 1 | 7 |
| Alene | 2014 | 4 | 2 | 1 | 7 |
| Gebre | 2015 | 4 | 2 | 2 | 8 |
| Weldekidan | 2018 | 4 | 2 | 2 | 8 |
| Kenea | 2018 | 4 | 2 | 2 | 8 |
| Abdella | 2020 | 4 | 2 | 1 | 7 |
| Tan | 2020 | 4 | 2 | 1 | 7 |
| Kedir | 2021 | 4 | 2 | 1 | 7 |
| Hailu | 2019 | 4 | 2 | 2 | 8 |
| Kejela | 2020 | 4 | 2 | 1 | 7 |
| Zerfu | 2019 | 4 | 2 | 2 | 8 |
| Bekele | 2016 | 4 | 2 | 2 | 8 |
| Belay | 2019 | 4 | 2 | 2 | 8 |
| Anwary | 2021 | 4 | 2 | 2 | 8 |
| Noronha | 2010 | 4 | 2 | 1 | 7 |
| Abdu | 2021 | 4 | 2 | 2 | 8 |
| OBORO | 2002 | 4 | 2 | 2 | 8 |
| Woldegebriel | 2020 | 4 | 2 | 2 | 8 |
| Gari | 2020 | 4 | 2 | 2 | 8 |
| Deriba | 2020 | 4 | 2 | 2 | 8 |
| Ngimbudzi | 2021 | 4 | 2 | 2 | 8 |
| Balis | 2022 | 4 | 2 | 2 | 8 |
| Engmann | 2008 | 4 | 2 | 2 | 8 |
| Asrie | 2017 | 4 | 2 | 1 | 7 |
| Okia | 2019 | 4 | 2 | 2 | 8 |
| Debella | 2021 | 4 | 2 | 2 | 8 |
| Osman | 2020 | 4 | 2 | 2 | 8 |
| Teshome | 2020 | 4 | 2 | 2 | 8 |
| Tadesse | 2017 | 4 | 2 | 1 | 7 |
| ALRESHIDI | 2021 | 4 | 2 | 2 | 8 |
| Berhe | 2019 | 4 | 2 | 2 | 8 |
| Nonterah | 2019 | 4 | 2 | 2 | 8 |
| Lebso | 2017 | 4 | 2 | 2 | 8 |
| Tulu | 2019 | 4 | 2 | 1 | 7 |
| Gudeta | 2019 | 4 | 2 | 2 | 8 |
| Fondjo | 2020 | 4 | 2 | 2 | 8 |
| Azhar | 2021 | 4 | 2 | 1 | 7 |
| Obai | 2016 | 4 | 2 | 2 | 8 |
| Lin | 2018 | 4 | 2 | 2 | 8 |
| Grum | 2018 | 4 | 2 | 1 | 7 |
| Derso | 2017 | 4 | 2 | 2 | 8 |
| Kebede | 2018 | 4 | 2 | 2 | 8 |
| Berhe | 2019 | 4 | 2 | 2 | 8 |
| Zillmer | 2017 | 4 | 2 | 2 | 8 |
